# Understanding the function of regulatory DNA interactions in the interpretation of non-coding GWAS variants

**DOI:** 10.3389/fcell.2022.957292

**Published:** 2022-08-19

**Authors:** Wujuan Zhong, Weifang Liu, Jiawen Chen, Quan Sun, Ming Hu, Yun Li

**Affiliations:** ^1^ Biostatistics and Research Decision Sciences, Merck & Co, Inc, Rahway, NJ, United States; ^2^ Department of Biostatistics, University of North Carolina at Chapel Hill, Chapel Hill, NC, United States; ^3^ Department of Quantitative Health Sciences, Lerner Research Institute, Cleveland Clinic Foundation, Cleveland, OH, United States; ^4^ Department of Genetics, University of North Carolina at Chapel Hill, Chapel Hill, NC, United States; ^5^ Department of Computer Science, University of North Carolina at Chapel Hill, Chapel Hill, NC, United States

**Keywords:** 3D genome organization, GWAS variants, non-coding DNA variation, Hi-C, TADs, FIREs, chromatin interactions

## Abstract

Genome-wide association studies (GWAS) have identified a vast number of variants associated with various complex human diseases and traits. However, most of these GWAS variants reside in non-coding regions producing no proteins, making the interpretation of these variants a daunting challenge. Prior evidence indicates that a subset of non-coding variants detected within or near cis-regulatory elements (e.g., promoters, enhancers, silencers, and insulators) might play a key role in disease etiology by regulating gene expression. Advanced sequencing- and imaging-based technologies, together with powerful computational methods, enabling comprehensive characterization of regulatory DNA interactions, have substantially improved our understanding of the three-dimensional (3D) genome architecture. Recent literature witnesses plenty of examples where using chromosome conformation capture (3C)-based technologies successfully links non-coding variants to their target genes and prioritizes relevant tissues or cell types. These examples illustrate the critical capability of 3D genome organization in annotating non-coding GWAS variants. This review discusses how 3D genome organization information contributes to elucidating the potential roles of non-coding GWAS variants in disease etiology.

## Introduction

Genome-wide association studies (GWAS) have achieved great success during the last two decades, reproducibly identifying hundreds of thousands of genetic variants associated with complex human diseases and traits (Buniello et al., 2019). However, only a small proportion (<10%) of GWAS variants alter the coding sequence of the human genome, where relatively straightforward hypotheses can be formed to link these variants to organism-level phenotypes directly. The remaining vast majority (i.e.,>90%) of GWAS variants reside in non-coding regions, making the interpretation of these variants a daunting challenge in the post-GWAS era (Hindorff et al., 2009; Sun et al., 2022).

To better understand the functional roles of non-coding GWAS variants, it is essential to annotate the non-coding regions, which account for ∼97% of the human genome. In recent years, the ENCODE consortium (ENCODE Project Consortium, 20l2; ENCODE Project Consortium et al., 2020) and the Roadmap Epigenomics Consortium (Roadmap Epigenomics Consortium et al., 2015) have identified millions of cis-regulatory elements (CREs) (including enhancers, promoters, silencers, and insulators) across a large number of human tissues and cell types. These CREs play critical roles in regulating the expression of their target genes in a cell-type-specific manner. Intriguingly, many studies have demonstrated significant enrichment of non-coding GWAS variants within CREs (Degner et al., 2012; Trynka et al., 2013; Zhang and Lupski, 2015), suggesting an indirect yet crucial role of these non-coding GWAS variants. Instead of directly changing the protein-coding DNA sequences, these non-coding variants may disrupt the functional roles of CREs, resulting in dysregulation of relevant genes.

The comprehensive annotation of CREs is a substantial step forward in understanding the non-coding GWAS variants. However, it remains challenging to assign non-coding GWAS variants-overlapped CREs to their target genes in disease-relevant tissues and cell types. How CREs regulate the expression of their target genes is still an open question in the genomics field. The difficulties lie in at least four aspects. First of all, the same CRE, such as a super-enhancer, may regulate multiple genes simultaneously. In addition, genes with critical functional roles, such as cell-type-marker genes, may be regulated by multiple CREs simultaneously to allow for some buffer in the presence of disrupted CREs. Along the line, we have recently reported super interactive promoters (SIPs) that interact with a more significant number of CREs than non-SIPs (Wen et al., 2022). Moreover, both the function of CREs and the relationship between CREs and their target gene(s) are highly tissue- or cell-type-specific. Last but not least, the majority of genes are not regulated merely by CREs in a close one-dimensional (1D) vicinity. Instead, CREs can form DNA loops with the promoter of their target gene(s) and regulate the expression of gene(s) from hundreds of kilobase (Kb) away (Dekker et al., 2013) or even over 1 Mb away (Fulco et al., 2016). Thus, a deep understanding of chromatin spatial organization can shed novel insights on gene regulation mechanisms and disease etiology.

Recently developed genomics and high-resolution imaging technologies (Jerkovic and Cavalli, 2021) provide revolutionary tools to map the nucleus’s three-dimensional (3D) genome. Coupling with powerful genome or epigenome editing tools such as CRISPR/Cas9, CRISPRi, and CRISPRa (Yin et al., 2017; Nakamura et al., 2021), researchers can not only measure the spatial proximity between non-coding GWAS variants-overlapped CREs and their putative target gene(s) but also functionally validate the role of CREs in disease-relevant cell types. For example, recent studies have shown that non-coding GWAS variants can alter the 3D chromatin structure and contribute to the risk of various disorders, including cancer, asthma, thalassemia, sex reversal, and limb malformation (Benko et al., 2011; Lupiáñez et al., 2015; Lupiáñez et al., 2016; Franke et al., 2016; Krijger and de Laat, 2016; Schmiedel et al., 2016; Schmitt et al., 2016b; Yu and Ren, 2017; Li et al., 2018; Liu et al., 2022b). Thus, characterizing 3D chromatin structure has the potential to prioritize disease causal genes, particularly those spatially close but far away in 1D genomic distance from their CREs, and reveal mechanistic insights underlying non-coding GWAS variants.

This review paper will describe the state-of-the-art experimental technologies, including sequencing-based and imaging-based approaches, to map chromatin spatial organization. In addition, we will summarize advanced computational methods to integrate transcriptome, epigenome, and 3D genome data to achieve a deep understanding of the functional roles of non-coding GWAS variants. We highlight recent breakthroughs in predicting and validating disease causal genes of non-coding GWAS variants and discuss challenges and opportunities for future endeavors.

## Experimental methods for detecting regulatory DNA interactions

**FIGURE 1:**
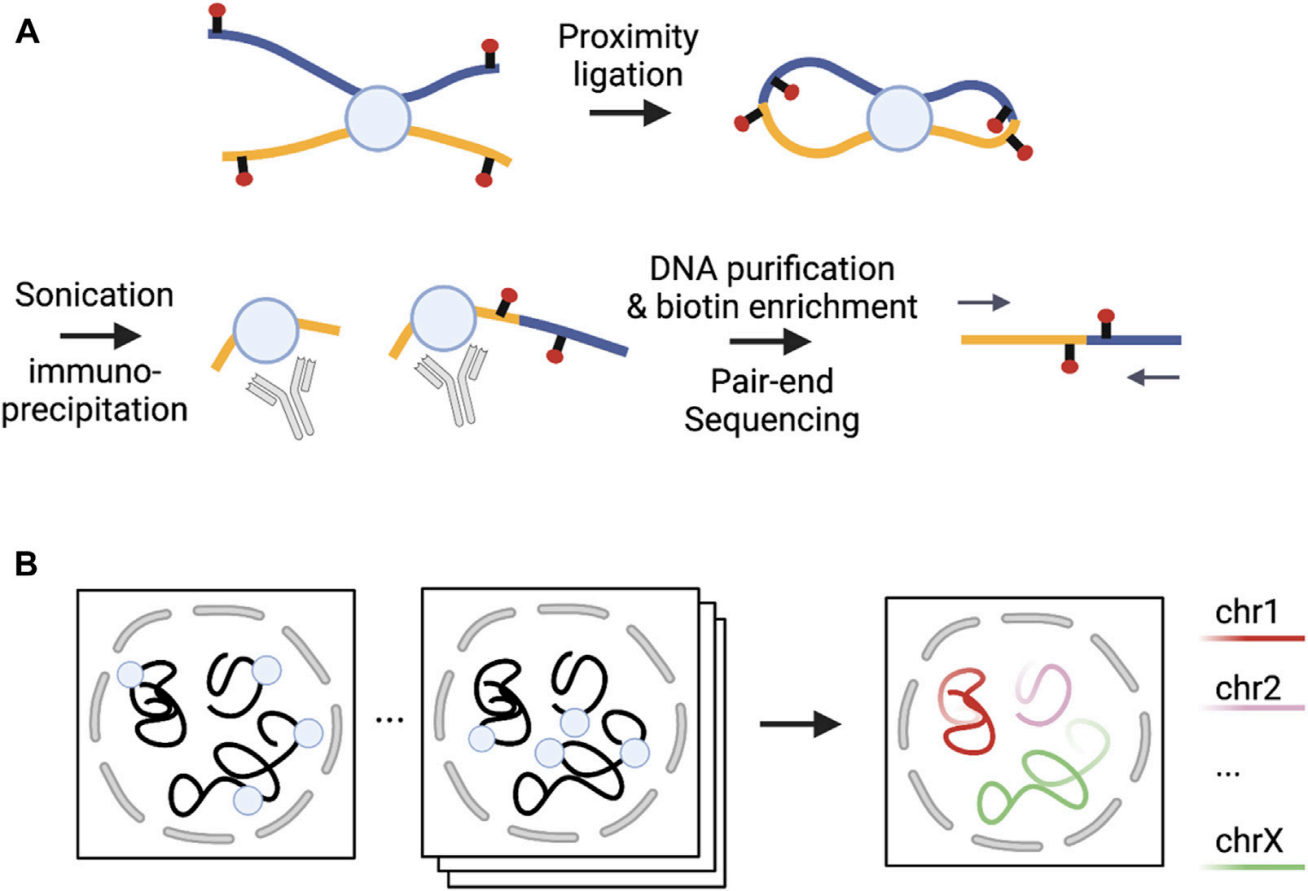
Illustrations of sequencing- and microscopy-based methods. **(A)** [Adapted from Figure 1A in (Fang et al., 2016)] Sequencing-based PLAC-seq method captures chromatin interactions mediated by a protein of interest; **(B)** [Adapted from Figure 1A in (Su et al., 2020)] Microscopy-based DNA MERFISH method allows multiplexed genome-scale imaging. Each square on the left of the arrow represents one round of imaging where each circle represents one locus imaged. In each round, multiple loci are simultaneously imaged. After many rounds of imaging, genome-scale imaging can be obtained. Note that the number of rounds required to image the same number of loci is inversely proportional to the number of loci imaged simultaneously, with substantially reduced number of rounds compared to the sequencing imaging strategy where only one locus is imaged in each round.

There are three major approaches for examining 3D genome structure: microscopy (imaging)-based techniques, sequencing-based approaches, and integrative methods (Figure 1). Microscopy-based approaches quantify cell-to-cell variability in chromatin architecture at certain genomic regions by visualizing the relative placement of these genomic regions in single cells (Jerkovic and Cavalli, 2021). In contrast, sequencing-based approaches measure chromatin contacts by crosslinking spatially close DNA segments and then applying deep sequencing to these crosslinked segments (Jerkovic and Cavalli, 2021). Integrative methods simultaneously leverage both sequencing- and microscopy-based methods, applying these two techniques to the same cell (Boninsegna et al., 2022).

Microscopy-based methods, including fluorescence *in situ* hybridization (FISH) and more advanced FISH-based techniques, estimate the relative distance by hybridizing DNA probes of specific genomic regions and then using a microscope for visualization (Su et al., 2020; Jerkovic and Cavalli, 2021; Zhuang, 2021). The earlier FISH-based methods were limited by the resolution and coverage of the genome. In terms of resolution, FISH-based methods have been significantly improved via the super-resolution microscopy technology that has increased spatial resolution. Regarding genome coverage, oligopaints-based FISH methods have been developed, where the oligopaints are fluorescently-labeled DNA oligonucleotides designed for imaging genomic regions (Beliveau et al., 2012, 2014). These methods include multiplex FISH (Zhuang, 2021) and OligoSTORM (Beliveau et al., 2017). Multiplex FISH can detect a larger number of loci by running multiple rounds of imaging fluorophore-labeled oligo probes—within each round, using different fluorophores for different regions to detect chromatin interactions. OligoSTORM is coupled with the STORM imaging technology for super-resolution imaging and can be further combined with other methods to increase coverage. Oligopaint barcode-based methods have been developed to increase further the efficiency of detecting chromatin interactions. These methods include the FISH-based ORCA method (Mateo et al., 2019) and OligoFISSEQ (Nguyen et al., 2020). ORCA partitions the target region into consecutive short regions with unique barcodes, where the barcodes are connected to probes carrying a common fluorophore-labeled oligo for imaging, avoiding the use of different fluorophores. OligoFISSEQ uses the FISSEQ technology (Lee et al., 2015) to read the oligopaints barcode for imaging and image multiple target regions for thousands of cells to estimate cell-to-cell variability. OligoFISSEQ can also be combined with OligoSTORM to image hundreds of target regions (Nguyen et al., 2020).

Sequencing-based methods can be categorized based on whether they can estimate chromatin interactions across the whole genome and implement proximity ligation to process crosslinked segments (Jerkovic and Cavalli, 2021). Under the former classification, methods covering the entire genome are non-enrichment methods, while methods covering specific types of interactions are enrichment methods. With the latter taxonomy, proximity ligation methods are C-based and otherwise non-C-based. Among non-enrichment methods, C-based methods such as Hi-C (Lieberman-Aiden et al., 2009) and its variants [e.g., Micro-C (Hsieh et al., 2016)] can generate all possible pairwise interactions of the whole genome. Unbiased Hi-C approaches require ultra deep sequencing depth for high resolution inference, which can be cost prohibitive. For example, we typically need several billion raw reads to detect chromatin interactions at Kb resolution. Non-C-based methods such as SPRITE (Quinodoz et al., 2018) and GAM (Beagrie et al., 2017) were developed using ligation-free technologies that allow for multi-way interactions. SPRITE quantifies higher-order chromatin interactions by adopting a split-pool approach to barcode the crosslinked DNA segments. In contrast, GAM maps spatial proximity of multiple DNA segment by determining the extent of co-segregation in the same cryo-sectioned and laser-microdissected compartment. While non-enrichment-based methods provide an unbiased view of the entire genome, enrichment methods have been proposed to empower closer and finer-resolution interrogation at interactions enriched in specific genomic regions or associated with particular proteins or epigenomic marks. The most commonly used C-based enrichment methods that do not involve probe design include ChIA-PET (Fullwood et al., 2009), HiChIP (Mumbach et al., 2016) and PLAC-seq (Fang et al., 2016). ChIA-PET (chromatin interaction analysis by paired-end tag) estimates interactions mediated by a protein of interest by first applying immuno-precipitation to enrich fragments with the protein of interest, and then the regular Hi-C proximity ligation before sequencing ligation products. In contrast, HiChIP and PLAC-seq technologies apply segmentation and proximity ligation first and then use protein immunoprecipitation for the enrichment of the desired ligation products. Capture-C (Hughes et al., 2014; Davies et al., 2016) and capture Hi-C (Mifsud et al., 2015) are also C-based enrichment methods. Compared to HiChIP and PLAC-seq, Capture-C and capture Hi-C require designing probes for a given set of sequences of interest (e.g., promoters or GWAS loci) to enrich ligation products in local regions. Among non-C-based enrichment methods, adapted-DamID (Cléard et al., 2006) first tethers DNA adenine methyltransferase (Dam) to a specific region and then detects DNA methylation patterns for this region and distant regions to identify chromatin interactions (Aughey et al., 2019).

Imaging-based and sequencing-based methods, as two orthogonal types of experimental approaches, have their own unique strengths and weaknesses. The key advantage of the imaging-based methods is to record 3D coordinates of each genomic locus, and directly measure spatial distance among genomic loci. In addition, imaging-based methods can achieve single cell resolution, facilitating the characterization of cell-to-cell variability in chromatin spatial organization. However, currently available imaging-based methods cannot yet simultaneously achieve Kb resolution and genome-wide coverage: existing methods either measure the whole genome at megabase (Mb) resolution (Takei et al., 2021a; 2021b), an entire chromosome or several Mb regions at 25–50 Kb resolution (Su et al., 2020; Takei et al., 2021a, 2021b), or a small region (∼210 Kb containing TSS of a gene of interest and its interacting enhancers) at 5 Kb resolution (Huang et al., 2021). It is still technically challenging to image the whole genome at Kb resolution, limiting its utility for genome-wide high resolution mapping of enhancer-promoter interactions in mammalian genomes.

In contrast, sequencing-based methods can generate Kb (Rao et al., 2014a; Bonev et al., 2017) or even nucleosome resolution (Krietenstein et al., 2020) map of mammalian 3D genomes, as long as the sequencing depth is sufficiently high. They usually enjoy higher sensitivity than imaging-based methods in terms of detecting genome-wide regulatory DNA interactions. One key weakness of sequencing-based methods is that they do not directly measure the spatial distance between genomic loci of interest, but rather gauge the frequency of the loci coming in spatial proximity, which is an indirect measure of 3D distance. Moreover, most widely used sequencing-based methods are designed for bulk samples containing 10^5^–10^6^ cells. Single-cell-based sequencing methods, including single-cell Hi-C (scHi-C) (Nagano et al., 2013), sci-Hi-C (Kim et al., 2020), sc-m3c-seq (Lee et al., 2019) and Dip-C (Tan et al., 2018), all suffer from limited capture efficiency per cell, making the quantification of cell-to-cell variability extremely challenging.

Taken together, investigators need to balance the pros and cons of different experimental methods, based on their specific scientific questions. For example, we would recommend imaging-based methods when the primary interest is to understand cell-to-cell variability in regulatory DNA interactions near a specific gene or element of interest. While for another example, when the primary goal is to comprehensively characterize genome-wide enhancer-promoter interactions, sequencing-based methods would be a better choice.

Integrative approaches have been developed to combine the advantages of imaging- and sequencing-based methods for better genome coverage and higher resolution. For example, *in situ* genome sequencing (IGS) was designed to jointly conduct sequencing and imaging simultaneously for intact genomes and directly link DNA sequence to 3D spatial proximity (Payne et al., 2021). However, IGS does not allow an adequate evaluation of enhancer-promoter interactions due to the limited resolution. Other integrative methods are comprehensively reviewed by Boninsegna et al. (2022).

## Utilizing 3D genome architecture to interpret disease-related genetic variants

Advanced technologies for studying 3D genome organization have generated an increasing amount of useful data. Accompanying advances in computational methods have enabled detection and quantification of various layers of chromatin spatial organization, including topologically associating domains (TADs) (Dixon et al., 2012; Crane et al., 2015; Rocha et al., 2015; Dali and Blanchette, 2017; Forcato et al., 2017; Zufferey et al., 2018; Liu et al., 2022a; Sefer, 2022), frequently interacting regions (FIREs) (Schmitt et al., 2016a; Crowley et al., 2021), and chromatin interactions (Ay et al., 2014; Rao et al., 2014b; Xu et al., 2016a, 2016b; Carty et al., 2017; Forcato et al., 2017; Cao et al., 2020; Kaul et al., 2020; Roayaei Ardakany et al., 2020; Rowley et al., 2020; Lagler et al., 2021; Sahin et al., 2021; Yu et al., 2021) (Table 1). These valuable pieces of 3D genome architecture information have been widely used to identify candidate risk genes for non-coding GWAS variants associated with complex diseases (Smemo et al., 2014; Giorgio et al., 2015; Schmitt et al., 2016a; Lupiáñez et al., 2016; Won et al., 2016; Martin et al., 2017; Fulco et al., 2019; Crowley et al., 2021; Yu et al., 2021). For instance, disarrangement of TAD boundaries can disrupt normal regulatory architecture and possibly form new loops, resulting in gene dysregulation, eventually leading to phenotypic aberrations (Lupiáñez et al., 2015; Krijger and de Laat, 2016). At the FIRE level, overlapping GWAS variants with FIREs can help to prioritize causal variants among many of their linkage disequilibrium (LD) tags (Huang et al., 2022a) and subsequently prioritize the putative effector genes in the neighborhood of FIREs in a tissue- or cell-type-specific manner (Schmitt et al., 2016a). At the most refined chromatin loop/interaction level, interruption of enhancer-promoter interactions can alter gene expression to cause diseases (Smemo et al., 2014; Krijger and de Laat, 2016). Finally, integrative approaches combine data from multiple resources to interpret non-coding variants, such as integrating chromatin structure information with other omics data to identify significant chromatin interactions, ensembling sequencing- and imaging-based data to simulate 3D genome structures, as reviewed in Liu et al. (2022b) and Boninsegna et al. (2022).

**TABLE 1:** Review papers and collections of computational approaches for chromatin interactions and domains.

Title	Category	Description	Year	References
A critical assessment of topologically associating domain prediction tools	TADs	Compared seven TAD calling methods	2017	Dali & Blanchette, (2017)
Comparison of computational methods for Hi-C data analysis	TADs and chromatin interactions	Compared seven TAD calling methods and six chromatin interaction callers	2017	Forcato et al. (2017)
Comparison of computational methods for the identification of topologically associating domains	TADs	Compared 20 TAD calling methods	2018	Zufferey et al. (2018)
Computational methods for analyzing genome-wide chromosome conformation capture data	General pipeline	Reviewed pipelines and methods for 3C-based data	2018	Nicoletti et al. (2018)
Computational methods for assessing chromatin hierarchy	General pipeline	Reviewed computational tools for assessing chromatin hierarchy	2018	Chang et al. (2018)
Computational methods for analyzing and modeling genome structure and organization	General pipeline	Reviewed analytic and modeling techniques for 3C-based methods	2018	Lin et al. (2019)
Hi-C analysis: from data generation to integration	General pipeline	Reviewed methods for Hi-C data analysis	2019	Pal et al. (2019)
Comparison of computational methods for 3D genome analysis at single-cell Hi-C level	General pipeline	Compared the performance of Hi-C methods on ultra-sparse Hi-C data	2020	Li et al. (2020)
Computational methods for the prediction of chromatin interaction and organization using sequence and epigenomic profiles	Prediction	Summarized 48 computational methods for predicting chromatin interactions and spatial organization features	2021	Tao et al. (2021)
A comparison of topologically associating domain callers over mammals at high resolution	TADs	Compared 27 TAD calling methods	2022	Sefer (2022)
A comparison of topologically associating domain callers based on Hi-C data	TADs	Compared 26 TAD calling methods	2022	Liu et al. (2022a)
Bacon: a comprehensive computational benchmarking framework for evaluating targeted chromatin conformation capture-specific methodologies	Chromatin interactions	Benchmarked 12 computational pipelines for HiChIP/PLAC-seq and/or ChIA-PET data	2022	Tang et al. (2022)
Hi-C data analysis tools and papers	General pipeline	A collection of Hi-C tools and papers	Accessed on 05/27/2022	https://github.com/mdozmorov/HiC_tools
4DN Software	General pipeline	A collection of data analysis and visualization tools for studying the 3D genome	Accessed on 05/27/2022	https://www.4dnucleome.org/software.html

We first review some examples using chromatin interactions to prioritize putative target genes. One of the earliest and most renowned examples was reported by Smemo et al. (2014), where the authors elegantly elucidated molecular mechanisms underlying the noncoding obesity-associated GWAS variants at the *FTO* locus with chromatin interactions identified from 4C-seq (van de Werken et al., 2012), a C-based method that quantifies chromatin spatial proximity between a specific region of interest and all genomic loci in its neighborhood. Specifically, long-range chromatin interactions link *FTO* intronic variants to their target gene *IRX3* (Smemo et al., 2014). Simultaneously considering long-range chromatin interactions, epigenetic annotations, and eQTL data, we can identify and prioritize causal variants and target genes for various human diseases and traits. Studies have shown that the majority of noncoding variants interact with distal genes based on Hi-C (Song et al., 2019; Sey et al., 2020), highlighting the importance of chromatin 3D organization in prioritization and functional follow-up of GWAS variants.

As the number and size of GWAS continue to grow rapidly, increasing evidence shows that regulatory variants function in a tissue- or cell-type-specific manner (Schmitt et al., 2016a; Barbeira et al., 2018; Gallagher and Chen-Plotkin, 2018; Sun et al., 2022). Literature in the past decade has accumulated many examples where long-range chromatin interactions have aided the prioritization and establishment of target genes for GWAS variants in disease-relevant tissues and cell types. For example, SnapHiC (Yu et al., 2021), the first computational method developed to identify chromatin interactions from single cell Hi-C data, reported long-range chromatin interactions between two GWAS variants (rs112481437 and rs138137383) associated with Alzheimer’s disease and *APOE*, specifically in astrocytes but not in other brain cell types. Other examples include a schizophrenia (SCZ) GWAS variant (rs1191551) forming a long-range (∼760 Kb away) interaction with the promoter of *FOXG1* revealed by fetal brain Hi-C data (Won et al., 2016); a long-range (>500 Kb away) interaction in liver between the promoter of *FST* and a type 2 diabetes (T2D)-associated SNP rs6450176, which is an intronic variant in *ARL15* (Martin et al., 2017); an interaction between the promoter of *BACH2* and rs72928038 (∼30 Kb away), an intronic variant in *BACH2* associated with various diseases including multiple sclerosis and type 1 diabetes, detected using promoter capture Hi-C data in naive CD4^+^ T cells (Kundu et al., 2022), and an interaction between the promoter of *GATA3* and rs3824662 (∼7 Kb), a *GATA3* intronic variant associated with Philadelphia chromosome-like childhood acute lymphoblastic leukemia (Yang et al., 2022). Such tissue- or cell-type-specific long-range chromatin interactions will greatly facilitate functional experiments, accelerating the uncovery of molecular mechanisms and new therapeutic targets.

**FIGURE 2:**
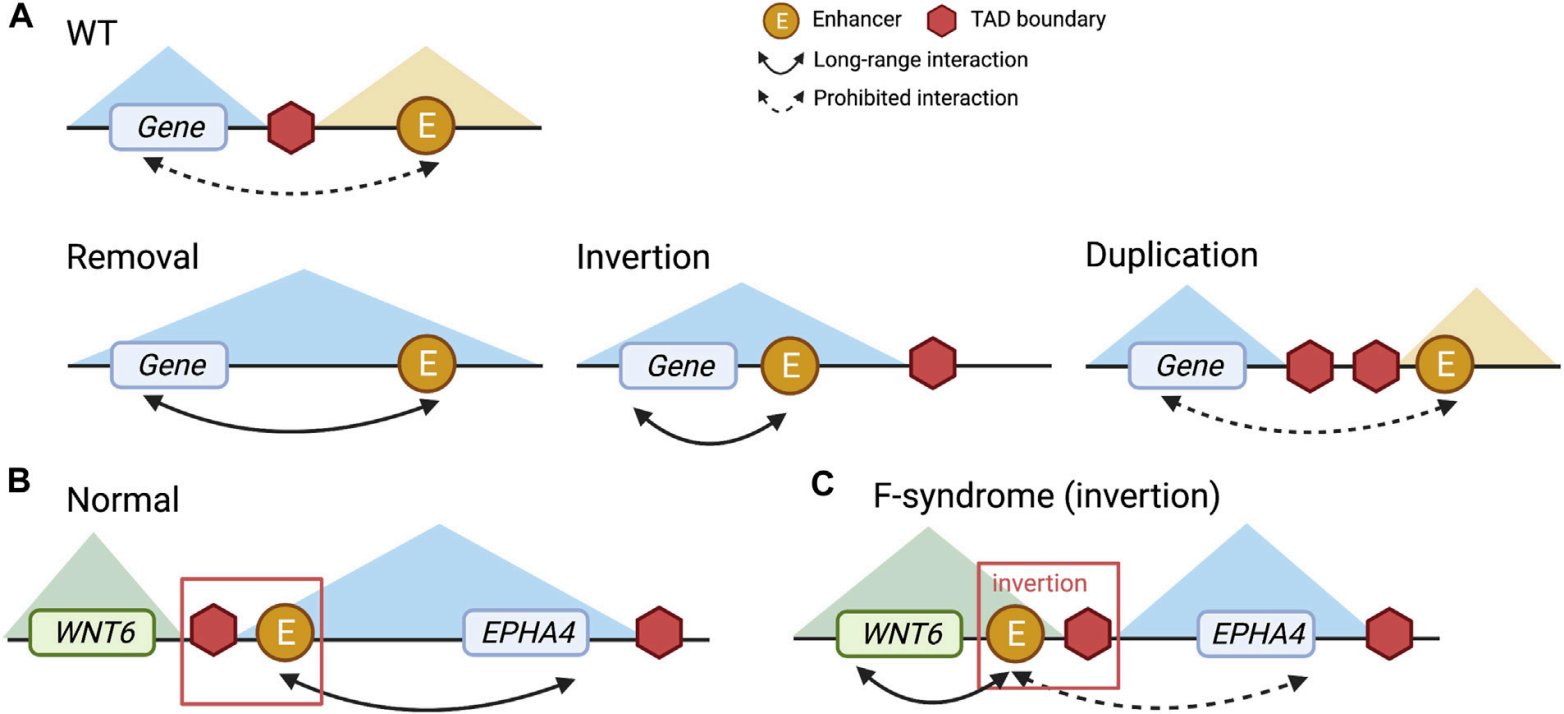
Different types of TAD boundary alteration and the *EPHA4* example. **(A)** Wild type (WT), removal, inversion, and duplication of TAD boundary. **(B)** The normal status of TAD boundaries at the *EPHA4* locus. **(C)** With an inversion genetic variant, aberrant TAD boundaries at the *EPHA4* locus were observed in F-syndrome patients. The enhancer and TAD boundary to the left of *EPHA4* are inverted, resulting in repression of *EPHA4* expression and activation of *WNT6* expression.

Next, we will review examples where TAD boundaries are disrupted by non-coding variations, which result in enhancer-promoter interaction changes. Specifically, impacts of non-coding variants on TADs include removing, inverting, and duplicating TAD boundaries. These changes can break regular links between enhancers and promoters present in wild type and create new links that do not exist otherwise (Figure 2A) (Yu and Ren, 2017). One example is at the *LMNB1* locus, where the deletion of a TAD boundary leads to an autosomal dominant, slowly progressive, and yet fatal adult-onset demyelinating leukodystrophy (ADLD) disorder. Specifically, the *LMNB1* gene becomes highly expressed due to the missing boundary leading to new chromatin interactions between the promoter of the *LMNB1* gene and several other enhancers (Giorgio et al., 2015; Yu and Ren, 2017). In another example, duplication and inversion of TAD boundaries near *EPHA4* and *WNT6* genes cause limb malformation. Specifically, as illustrated in Figure 2, disrupted TAD boundaries lead to significantly increased *WNT6* gene expression and decreased *EPHA4* gene expression (Figures 2B,C), resulting in syndactyly (Lupiáñez et al., 2015; Angier, 2017). Yu and Ren (2017) provide an excellent review, covering multiple examples where aberrations in TAD boundaries lead to phenotypic abnormalities. These studies demonstrate that genetic variations around TAD boundaries can modify expression patterns of nearby genes and illustrate the importance of studying alternations in the regulatory landscape through 3D genome structure (Figures 2B,C).

**FIGURE 3:**
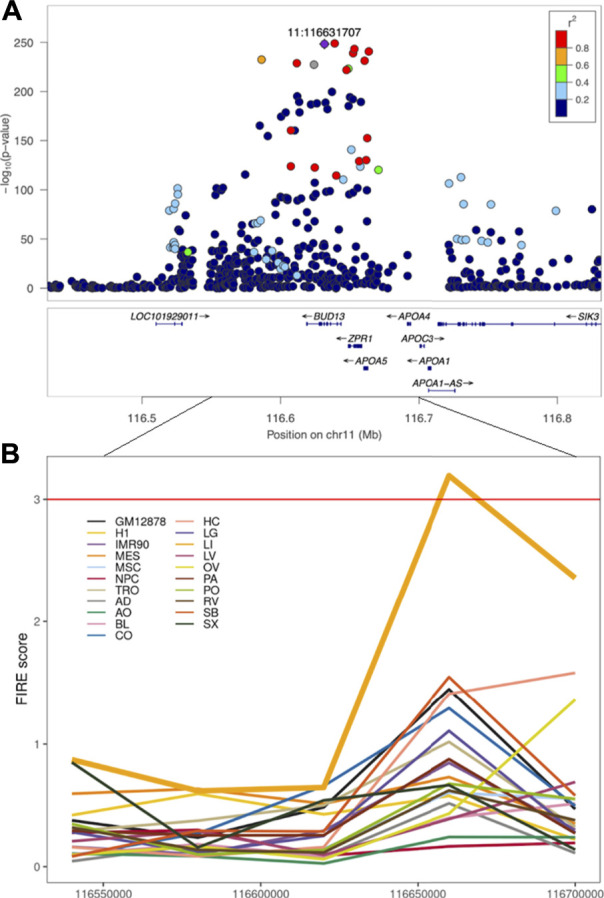
Triglycerides-GWAS signals near a liver-specific FIRE region. **(A)** Locuszoom plot of GWAS results for triglycerides (Willer et al., 2013). **(B)** FIRE scores across 21 human cell lines and primary tissues examined in Schmitt et al. Each color represents a tissue or cell line. GM12878: the GM12878 lymphoblastoid cell line (LCL), H1: the H1 human embryonic stem cell line, IMR90: the IMR90 human lung fibroblast cell line, MES: the human mesendoderm cell line, MSC: the human mesenchymal stem cell lines, NPC: the human neural progenitor cell line, TRO: the human trophoblasts-like cell line, AD: the human adrenal gland tissue, AO: the human aorta tissue, BL: the human bladder tissue, CO: the human dorsolateral prefrontal cortex tissue, HC: the human hippocampus tissue, LG: the human lung tissue, LI: the human liver tissue, LV: the human left ventricle tissue, OV: the human ovary tissue, PA: the human pancreas tissue, PO: the human psoas muscle tissue, RV: the human right ventricle tissue, SB: the human small bowel tissue, SX: the human spleen tissue.

Furthermore, we will introduce several examples using FIREs to prioritize causal variants and the tissues or cell types where the causal variants exert their effects. For instance, when overlapping triglycerides-GWAS variants (Willer et al., 2013) on chromosome 11 with FIREs across 14 human primary tissues and seven cell types, a liver-specific FIRE overlapped the region harboring GWAS variants (Figure 3) (Schmitt et al., 2016a). This observation suggested that liver is likely the tissue where the GWAS variants play functional roles. Although in this case liver was known to be highly relevant for lipid metabolism, this finding serves as a successful proof-of-concept where tissue- or cell-type-specific FIREs can help prioritize the most pertinent tissues or cell types. Other examples include an asthma-GWAS variant (rs755023315) (Han et al., 2020) residing in a GM12878-specific FIRE that overlaps with an immune-related gene *CD70* (Schmitt et al., 2016a) and a SCZ-GWAS variant (rs9960767) residing in a hippocampus super-FIRE overlapping with the neurodevelopment related gene *TCF4* (Crowley et al., 2021). Although more recently developed, FIREs have been recognized for their roles in annotating functions of non-coding variants due to their high tissue- or cell-type specificity.

**FIGURE 4:**
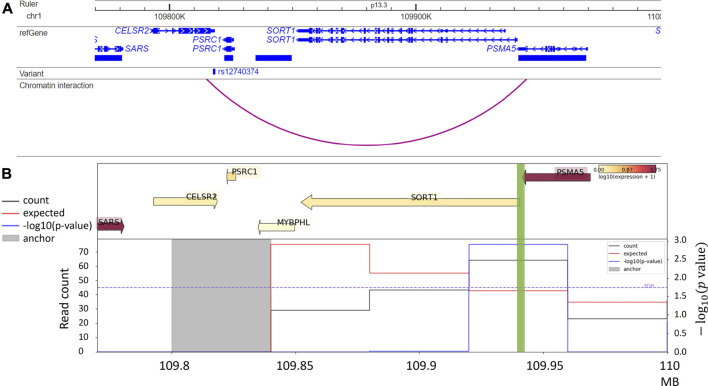
**(A)** Chromatin interaction between rs12740374, an LDL GWAS variant, and promoter of the *SORT1* gene, reported by Fulco et al. (2019); **(B)** Virtual 4C plot from HUGIn (Martin et al., 2017), for the same region in Panel A, shows a significant chromatin interaction between the anchor bin harbor rs12740374 (the gray highlighted region) the and the promoter of the *SORT1* gene (green highlight), in human liver tissue. The top panel shows gene expression levels and the bottom panel includes three lines quantifying chromatin interactions between the anchor bin and all other bins in the region: black line denotes the observed counts, red line denotes the expected counts, and blue line denotes the -log_10_ (*p* value).

In addition, target genes for GWAS variants can also be predicted from integrative analysis. For example, the Activity-by-Contact (ABC) model, combining chromatin activity and interaction information, assigns rs12740374, a GWAS variant associated with low-density lipoprotein cholesterol (LDL) to the *SORT1* gene. The authors additionally reported that this variant is a liver-eQTL for *SORT1* and further validated its impact on *SORT1* gene expression via CRISPR genome editing in primary hepatocytes (Fulco et al., 2019). We visualize the example in Figure 4A. Consistent with predictions by the ABC model, this chromatin interaction is also detected from liver Hi-C data (Schmitt et al., 2016a) with a significant interaction between the anchor bin (the gray highlighted region) including the GWAS variant rs12740374 and the bin containing the promoter of the *SORT1* gene (green highlight) (Figure 4B). The ABC model shows the possibility of using non-liver Hi-C data (K562 Hi-C data) with liver enhancer activity data (H3K27ac ChIP-seq data in liver tissue) to prioritize enhancer-promoter interactions in the liver (Fulco et al., 2019).

**FIGURE 5:**
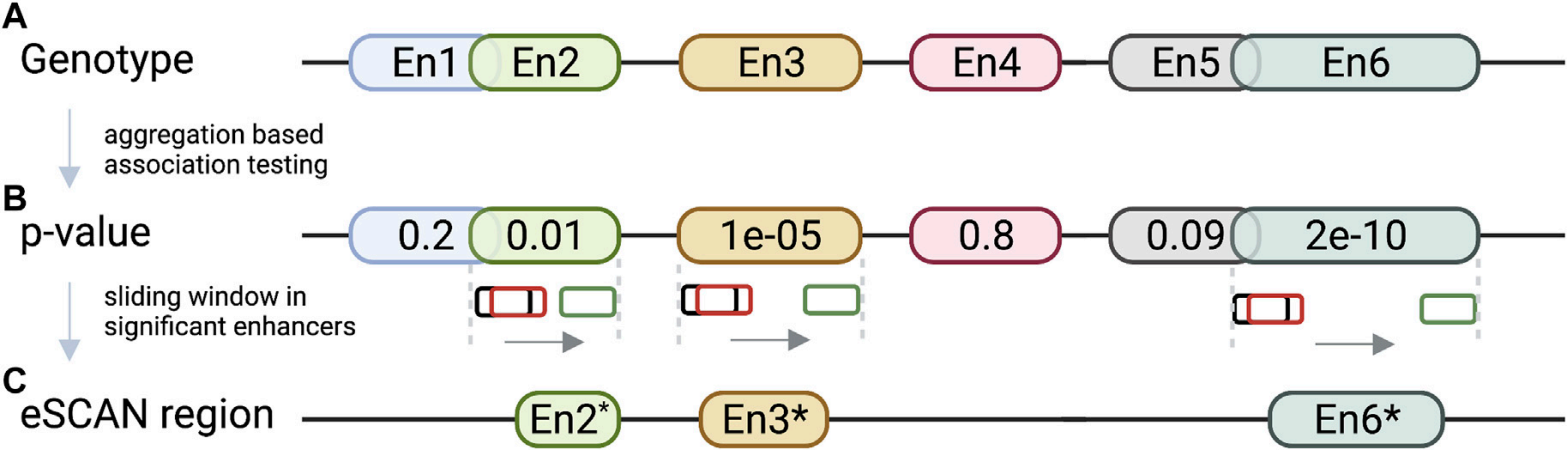
eSCAN workflow. **(A)** eSCAN takes genotype and phenotype as well as a list of predefined enhancer (En1-En6 in the illustration) regions as input. **(B)** Aggregation-based association tests are performed in the enhancer-screening step to identify significant enhancer(s). In this illustration, En2 (green), En3 (yellow), and En6 (turquoise) are deemed significant. **(C)** eSCAN performs dynamic sliding window scanning within the significant enhancer region(s) to further narrow down the associated region. For example, En2* is the associated sub-region within En2 after narrowing down via dynamic scanning. Similar for En3* and En6*.

In addition to the specific examples we described, many other studies have been conducted to understand whether and how non-coding variations exert their functions. For example, Figure 4B shows a virtual 4C plot using the HUGIn tool (Martin et al., 2017), which was developed to visualize chromatin interactions anchored at GWAS variants or regulatory regions of interest based on a compendium of Hi-C data from 14 primary human tissues and seven human cell lines. HUGIn tool also visualizes gene expression and epigenomic data, which can further facilitate researchers to prioritize target genes at GWAS loci. For another example, the E + G + Methyl (Wu and Pan, 2019) method performs a gene-based aggregation association test by integrating enhancer-promoter interactions and methylation QTLs with GWAS summary statistics. E + G + Methyl gains statistical power to detect target genes for GWAS variants by jointly modeling these two pieces of complementary information but the availability of both (i.e., enhancer–promoter interaction data and methylation QTL data) would limit the application of E + G + Methyl. In addition, because single-variant GWAS summary statistics are used for integration, rare variants would be under-represented in E + G + Methyl analysis. Applying E + G + Methyl to study SCZ, the authors identified several novel genes associated with SCZ, which standard GWAS missed. Along the same line, Yang et al. present the eSCAN method (Yang et al., 2022) (illustrated in Figure 5), an aggregation-based association testing framework that integrates various functional annotations, including chromatin accessibility, histone marks, and chromatin spatial organization. eSCAN uses these functional annotations to define “enhancers”, or more precisely, putative regulatory elements, and performs scanning across these putative enhancer regions. The scanning approach adopted by eSCAN allows simultaneous search and refinement of associated regions within the putative enhancers, using both genotype and phenotype data, rather than testing on *a priori* defined genes or region units. The eSCAN method focuses on variants residing in putative enhancer regions, which can increase statistical power by reducing the search space among non-coding regions. Furthermore, with its scan feature, eSCAN tends to identify associated regions that are shorter in size, effectively achieving fine-mapping of causal variants and regions. Integration with chromatin conformation data also makes easier biological interpretation of detected regions. As an aggregation method that tests a set of variants, eSCAN may not be able to narrow down to single variant level. With higher resolution (Kb or finer) chromatin conformation data, eSCAN can potentially pinpoint individual variants. When applied to hematological traits, eSCAN pinpointed multiplied regulatory regions associated with various blood cell indices. These regions were either missed by alternative methods or in much coarser resolution. Among them, a regulator region (chr6:90, 423, 754–90,425,200) was associated with platelet count, a signal missed by standard GWAS. The gene it regulates, the *BACH2* gene, is an essential immune cell regulatory factor and plays a critical role in maintaining regulatory T-cell function and B-cell maturation (Afzali et al., 2017). These methods, integrating epigenomic information, including chromatin conformation data in genetic association testing, allow discovery, refinement, and interpretation of regulatory regions associated with complex diseases and traits. We anticipate that these methods will lead to more exciting findings in the near future, particularly given chromatin conformation data accumulated in more tissues and cell types relevant to various diseases and traits.

## Discussion

Knowledge of genome-wide chromatin spatial organization has been significantly advanced, particularly since 2009, with the advent of Hi-C (Lieberman-Aiden et al., 2009) and Hi-C-derived technologies. We anticipate more rapid advancement and increasingly diverse data generated with the constantly evolving sequencing- and imaging-based technologies to study 3D chromatin structure (Liu et al., 2022b). These technologies enhance our understanding of chromatin 3D organization in general and arrive timely to help interpret GWAS findings, which have successfully identified hundreds of thousands of genetic variants associated with various diseases and traits (Buniello et al., 2019). These GWAS variants, easily reaching millions when including variants that are in LD (Huang et al., 2022a) with the index variants initially detected, reside predominantly in non-coding regions of the genome (Zhang and Lupski, 2015; Martin et al., 2017) with functional mechanisms remaining elusive. There is a pressing need to link GWAS variants to their target genes in disease-relevant tissues or cell types to advance these GWAS findings from variants to function (Sullivan and Susztak, 2020; Rowland et al., 2022b; Sun et al., 2022), to improved understanding of disease etiology, to the development of new drugs, and ultimately to personalized medicine.

Despite tremendous advances in both experimental technologies and computational methods to study chromatin spatial organization, multiple challenges and gaps remain before we can fully leverage DNA 3D organization information for the interpretation of GWAS results.

First, multiple layers of biases are buried in data generated from Hi-C and other C-based technologies. For Hi-C data, both explicit and implicit normalization methods have been developed to mitigate such biases. Explicit normalization assumes that systematic biases, due to restriction enzyme cutting frequency, GC content or sequence uniqueness (Yaffe and Tanay, 2011), are known *a priori*, and can be removed by explicit model-based approaches (Yaffe and Tanay, 2011; Hu et al., 2012). In contrast, implicit normalization methods such as ICE, VC and KRnorm (Imakaev et al., 2012; Rao et al., 2014b) assume the presence of unknown biases and perform normalization based on equal visibility assumption (Imakaev et al., 2012). Data generated from other C-based methods suffer from additional biases. For example, capture Hi-C data suffers from probe capture efficiency bias, while HiChIP and PLAC-seq data contain bias from immunoprecipitation efficiency. Reducing or removing biases from C-based as well as imaging data remains an active research area.

Second, we still need efficient and innovative methods to integrate chromatin interaction information with complementary pieces of information. Although we review multiple approaches and methods that leverage chromatin conformation data with various other sources of data (e.g., methylation QTL for E + G + Methyl, chromatin accessibility and histone marks for eSCAN), methods that integrate additional omics data at either bulk tissue or single cell level will further enhance power to prioritize and pinpoint important functional variants, regions and genes, and potentially in tissue- or cell-type-specific manner.

Third, studying of chromatin spatial organization can further benefit from advanced machine learning or deep learning methods. Deep learning-based methods have been used for chromatin interaction prediction or Hi-C and alike data enhancement. For example, Akita (Fudenberg et al., 2020) adopts a convolutional neural network (CNN) to predict chromatin interactions using DNA sequences alone, which can be leveraged to predict the regulatory potential of GWAS variants by assessing their impact on chromatin spatial organization. For another example, HiCPlus (Zhang et al., 2018) and HiCNN (Liu and Wang, 2019), both also CNN-based, have been proposed for the enhancement of Hi-C data and show promising results when applied to enhance HiChIP and PLAC-seq data (Huang et al., 2022b). With increasing scale and complexity of the data, we anticipate deep learning-based methods can further manifest their advantages to extract non-linear and complex relationships among high-dimensional features.

**FIGURE 6:**
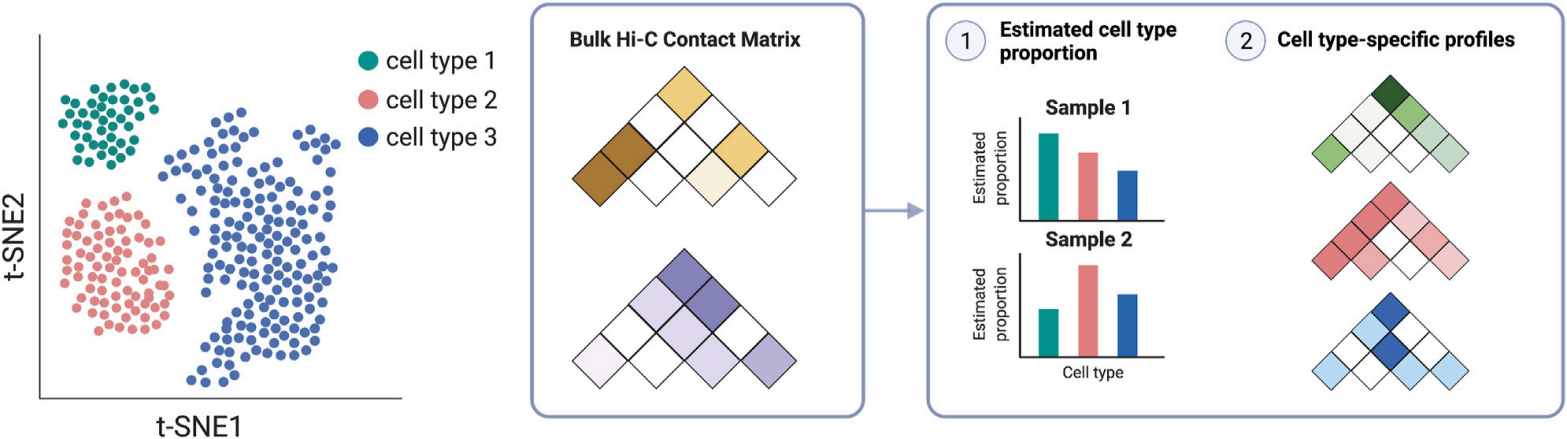
Cell deconvolution methods take bulk Hi-C contact matrices as input to infer cell-type proportion in each sample and cell-type-specific profiles.

Finally, as a community, we need to generate high quality, high resolution data from complementary technologies in diverse biosamples. First, we need more comprehensive compendia of chromatin conformation data. Such data holds and has been delivering on the promise of helping to fulfill the crucial variant-to-function task. Future efforts should encompass diverse tissues and cell types across developmental stages, multiple disease progression time-points, and under various natural and perturbed conditions, as provided by recent publications (Schmitt et al., 2016a; Jung et al., 2019; Song et al., 2019, 2020) and efforts within the 4D Nucleome Project (Dekker et al., 2017). Second, we need more single-cell data. Recent single-cell technologies (Zhou et al., 2021; Yu et al., 2022) have further enhanced our capabilities to characterize cell-type-specific profiles as well as to potentially reveal cell-to-cell variability, which will additionally facilitate our interpretation and understanding of GWAS results (Yu et al., 2021; Li et al., 2022). In addition, chromatin interactome profiles in population samples will also be essential to understanding the variation across individuals, the genetics behind the variation (Gorkin et al., 2019), and the consequence of such variation for the inference of the molecular causal paths via causal inference or mediation analysis (Zhong et al., 2019, 2022). Such multi-sample chromatin conformation data have emerged at the bulk level encompassing many cells (Gorkin et al., 2019; Chandra et al., 2021). Cell type deconvolution can be essential when analyzing multi-sample data from tissue samples to ensure valid inference and gain insights in a cell-type-specific manner (Figure 6) (Sefer et al., 2016; Rowland et al., 2022a). We anticipate future studies involving single-cell data, similar to multi-sample single-cell RNA-sequencing data (Ren et al., 2021; Zheng et al., 2021), which can provide insights into disease etiology at an even more refined resolution (van Buren et al., 2021, 2022; Zhang et al., 2022).

Interpretation of GWAS results has received extensive attention in the past two decades, with many alternative approaches proposed and employed to achieve the variant-to-function goal. For example, eQTL and co-localization with GWAS signals (GTEx Consortium, 2020; Kundu et al., 2022), transcriptome-wide association studies (Gamazon et al., 2015; Zhou et al., 2020; Wen et al., 2021; Tapia et al., 2022), and correlation between the epigenetic profile and expression of nearby gene(s) (Sheffield et al., 2013) are among the commonly adopted methods to identify target genes and relevant tissues and cell types for GWAS variants. Chromatin conformation data offers complementary information and has been found to enhance our capabilities in generating and prioritizing potential functional mechanisms when integrated with alternative approaches (Fulco et al., 2019; Marsha Wheeler et al., 2021; Sun et al., 2022). In addition, DNA 3D organization help us gain insights in the orchestration of different regulatory elements, revealing enhancer-enhancer networks (Beytebiere et al., 2019; di Giammartino et al., 2019), super enhancers that regulate multiple genes (Huang et al., 2018; Zhang et al., 2021), and super interactive promoters (Song et al., 2020; Wen et al., 2022) that tend to have higher extent of enhancer redundancy. We urge future studies to increasingly generate and leverage relevant chromatin 3D organization information, which will significantly facilitate advancing GWAS findings to ultimate clinical transformation.
